# Assessment of the potential respiratory hazard of volcanic ash from future Icelandic eruptions: a study of archived basaltic to rhyolitic ash samples

**DOI:** 10.1186/s12940-017-0302-9

**Published:** 2017-09-11

**Authors:** David E. Damby, Claire J. Horwell, Gudrun Larsen, Thorvaldur Thordarson, Maura Tomatis, Bice Fubini, Ken Donaldson

**Affiliations:** 1 US Geological Survey, Western Regional Offices, Menlo Park, CA USA; 2 Department of Earth and Environmental Sciences, Ludwig-Maximilians-Universität München, Munich, Germany; 3 Institute of Hazard, Risk and Resilience, Department of Earth Sciences, Durham University, Durham, UK; 4 Institute of Earth Sciences, Nordvulk, University of Iceland, Reykjavík, Iceland; 5 Dipartimento di Chimica, “G. Scansetti” Interdepartmental Center for Studies on Asbestos and other Toxic Particulates, Università degli Studi di Torino, Torino, Italy; 6 The Queen’s Medical Research Institute, The University of Edinburgh/MRC Centre for Inflammation Research, Edinburgh, UK

**Keywords:** Volcanic ash, Health hazard, Air pollution, Particle characterization, Free radicals, Haemolysis

## Abstract

**Background:**

The eruptions of Eyjafjallajökull (2010) and Grímsvötn (2011), Iceland, triggered immediate, international consideration of the respiratory health hazard of inhaling volcanic ash, and prompted the need to estimate the potential hazard posed by future eruptions of Iceland’s volcanoes to Icelandic and Northern European populations.

**Methods:**

A physicochemical characterization and toxicological assessment was conducted on a suite of archived ash samples spanning the spectrum of past eruptions (basaltic to rhyolitic magmatic composition) of Icelandic volcanoes following a protocol specifically designed by the International Volcanic Health Hazard Network.

**Results:**

Icelandic ash can be of a respirable size (up to 11.3 vol.% < 4 μm), but the samples did not display physicochemical characteristics of pathogenic particulate in terms of composition or morphology. Ash particles were generally angular, being composed of fragmented glass and crystals. Few fiber-like particles were observed, but those present comprised glass or sodium oxides, and are not related to pathogenic natural fibers, like asbestos or fibrous zeolites, thereby limiting concern of associated respiratory diseases. None of the samples contained cristobalite or tridymite, and only one sample contained quartz, minerals of interest due to the potential to cause silicosis. Sample surface areas are low, ranging from 0.4 to 1.6 m^2^ g^−1^, which aligns with analyses on ash from other eruptions worldwide. All samples generated a low level of hydroxyl radicals (HO^•^), a measure of surface reactivity, through the iron-catalyzed Fenton reaction compared to concurrently analyzed comparative samples. However, radical generation increased after ‘refreshing’ sample surfaces, indicating that newly erupted samples may display higher reactivity. A composition-dependent range of available surface iron was measured after a 7-day incubation, from 22.5 to 315.7 μmol m^−2^, with mafic samples releasing more iron than silicic samples. All samples were non-reactive in a test of red blood cell-membrane damage.

**Conclusions:**

The primary particle-specific concern is the potential for future eruptions of Iceland’s volcanoes to generate fine, respirable material and, thus, to increase ambient PM concentrations. This particularly applies to highly explosive silicic eruptions, but can also hold true for explosive basaltic eruptions or discrete events associated with basaltic fissure eruptions.

## Background

### Introduction

The 2010 eruption of Eyjafjallajökull volcano, Iceland, sent a plume of volcanic ash south-east across the Atlantic Ocean with ash fallout in Iceland, the UK and on mainland Europe [1]. Substantial, but localized, ashfall in Iceland triggered concerns and anxiety about the safety of inhaling volcanic particulate for the general population and susceptible groups [2, 3], since respirable particulate matter (PM) is known to negatively impact population health [4, 5] and has recently been classified as a carcinogen [6]. Although the thickness of ash deposited distally was negligible, the unanticipated phenomenon prompted similar public health concern across Northern Europe [7–9]. A year later, an eruption of Grímsvötn volcano produced minor ashfall in Iceland, the UK and Scandinavia [10, 11], again fueling the need to understand the respiratory hazard of these particles.

Physicochemical characteristics of inhaled particles are known determinants of biologic activity [12]. Therefore, predictive screens for assessing particle toxicity can help improve risk management advice [13]. The International Volcanic Health Hazard Network (www.ivhhn.org) promptly launched such efforts after both eruptions using a protocol designed to rapidly estimate the respiratory hazard through physicochemical and toxicological assessment of the ash [8, 14]. This protocol has been successfully implemented for a number of eruptions worldwide to inform ash hazard [8, 15–17]. The Eyjafjallajökull and Grímsvötn studies found that ash from the two eruptions did not possess most of the physicochemical characteristics often associated with acute or chronic respiratory disease, such as abundant crystalline silica or fiber-like particles. However, the ash from both eruptions contained respirable-sized (< 4 μm) material [8, 18], and caused a sustained pro-inflammatory response in lung cells in vitro [8], thereby indicating that inhaled material may trigger adverse health consequences.

In Iceland, the abundance of fine material in Eyjafjallajökull ash contributed significantly to ambient PM, due to both direct ash emissions [1] and re-suspension [19, 20], and several medical studies were initiated to determine any respiratory health consequences resulting from exposure to the ash. Early reported symptoms of upper airway and eye irritation and exacerbation of pre-existing asthma were limited, but ash exposure was associated with elevated prevalence of respiratory irritation and cough amongst residents of the most ash-exposed rural area close to Eyjafjallajökull [3, 21], and high PM levels were associated with emergency hospital visits in the capital city Reykjavík despite no ash accumulation [22]. In the immediately affected areas, an association was found between long-term exposure to volcanic ash and a higher prevalence of respiratory ailments [23], supporting the few previous epidemiological studies conducted elsewhere that consider moderate to heavy long-term exposure to ash and respiratory health [24].

Less is known about the health effects from exposure to long-range transported volcanic ash, although some cases of exacerbated respiratory ailments have been reported [25, 26]. As in Iceland, studies were prompted across Europe following the eruptions of Eyjafjallajökull and Grímsvötn due to the potential hazard posed. Increases in ambient PM were reported in Scandinavia but with inconclusive or unreported health outcomes [11, 27]. In the UK, ash was detected from both eruptions [10, 28], but syndromic surveillance did not pick up any adverse effects following ash exposure [7].

The Health Protection Agency (now Public Health England) carried out a review of the potential effects of volcanic ash inhalation, focusing on the low exposures likely to affect the UK [29], as part of work to inform UK policy during future ashfall events. This review occurred concurrently with the inclusion of the risk description for long-range volcanic hazards in the UK National Risk Registry of Civil Emergencies. The HPA, in consultation with IVHHN, recommends communicating the proportion of respirable material in ash and the presence or absence of crystalline silica at the earliest stage following a major eruption to public health officials so that they can provide evidence-based advice to the general public. The present study is a result of the recommendations from the aforementioned HPA review and aims to provide a baseline for early evidence on the likely physicochemical characteristics of different types of Icelandic ash, upon which initial advice during the next Icelandic ashfall incident can be founded.

The Icelandic volcanoes span a spectrum of eruption styles and magma types, from those that produce effusive, gas and lava-rich eruptions to those generating large explosions where vast quantities of ash are produced [30]. However, historical records and reconstructions from the geological record indicate that three quarters of all Holocene eruptions in Iceland were explosive [30, 31]. Cryptotephras (ash horizons in distal sedimentary sequences) have been identified across northwestern Europe from many of these eruptions [32], and there is evidence that the Icelandic volcanoes are entering a period of increased volcanic activity [33]. Therefore, there is an urgent need to assess the potential respiratory hazard of Icelandic ash from the range of volcanoes that might erupt in the foreseeable future, likely affecting communities in Iceland, the UK and the rest of Europe.

Due to the limited number of recent eruptions, compiling evidence on the effects that future eruptions may have on public health is best investigated through studying previous activity of the range of Icelandic volcanoes. In this study, we apply the screening strategy used on the Eyjafjallajökull 2010 and Grímsvötn 2011 ash [8] to samples of archived Icelandic ash, as analysis consistent with this protocol facilitates comparisons with existing heath studies. Only the particle-specific hazard is addressed herein. The recent Bárðarbunga-Veiðivötn fissure eruption at Holuhraun has highlighted how volcanic gas (predominantly SO_2_) and sulfate aerosol emissions can negatively impact air quality and health [34, 35], and previous eruptions of Iceland’s volcanoes have resulted in substantial environmental loading of adsorbed materials, particularly fluorine [36]. Both gas and leachate hazards also need to be considered during future eruptions.

### Geological setting

Postglacial volcanism in Iceland occurs within geographically distinct fissure systems and the associated central volcanoes, collectively termed volcanic systems [37, 38]. There are about 30 active volcanic systems in Iceland, 20 of which feature fissure swarms (fracture zones where eruptions occur along fissures) and 19 have at least one central volcano [39]. Felsic (silica rich) and intermediate magma is confined to the central volcanoes, whereas mafic (silica poor) magma has erupted at both the associated fissure swarms and at the central volcanoes [37, 38]. A total of 16 volcanic systems have been active in the last 11 centuries. Eruption frequency varies, being highest at the Grímsvötn volcanic system, with about 70 eruptions during this period. However, of the roughly 200 recorded eruptions, less than 30 erupted felsic or intermediate magma and only Askja, Hekla and Öræfajökull have produced dacitic or rhyolitic tephra [39, 40]. Iceland’s volcanic systems exhibit a variety of eruption styles, each broadly dependent on magmatic composition (i.e., basaltic to rhyolitic) and a system’s proximity to, and relationship with, surface waters, groundwaters and ice caps.

**Fig. 1:**
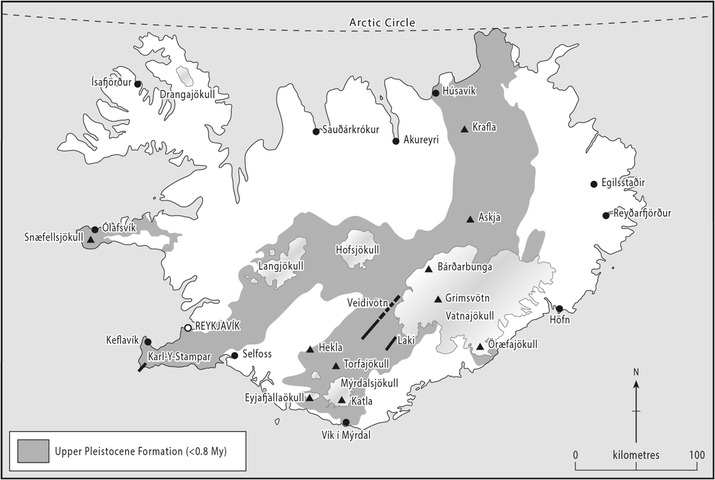
Locations of volcanic systems in Iceland relevant to this study. Central volcanoes/domains of volcanic systems from which ash was sourced are indicated by triangles or by a short line in case of large fissure eruptions. Also shown are towns (black or white circles) and ice caps (gray-white gradient-shaded areas). The Upper Pleistocene formation boundary (dark gray shaded area) is adapted from Thordarson and Höskuldsson [30]

Each volcano has distinctive eruption characteristics and products, and particles from different sources could have different toxicological effects. Therefore, a single hazard assessment cannot be made. However, the compositional trends of the products from each volcanic system have remained relatively stable throughout the Holocene, and many of them are geochemically distinct from other systems [37]. Due to the stability of the characteristics, physicochemical analysis of eruptive products from the Holocene can provide insight into the respiratory hazard posed by Iceland’s volcanoes during future eruptions. To consider this breadth of erupted products, 14 tephra samples were sourced from 11 eruptions at 8 central volcanoes for detailed investigation. These include the largest tephra layers produced by each of the relevant volcanic systems as eruptions of similar magnitudes would be the most likely to affect Iceland and the rest of Europe. The details of each sample are outlined in Table 1, and the location of each system is shown in Fig. 1.

**Table 1:** Sample and collection information for the volcanic ash samples analyzed in this study

Sample	Volcano/system	Eruption year	Collection date	Collection location	Type of activity
Askja-55d	Dyngjufjöll/Askja	1875	--	North rim of Öskjuvatn	phreatoplinian
Askja-56a	Dyngjufjöll/Askja	1875	--	North rim of Öskjuvatn	phreatoplinian
Askja-59b	Dyngjufjöll/Askja	1875	--	North rim of Öskjuvatn	phreatoplinian
Hekla-4	Hekla/Hekla	4.2 ka BP	23/07/1993	30 km NE of top crater	phreatoplinian
Hekla-1158	Hekla/Hekla	1158	29/10/1990	28 km NE of top crater	Plinian
Hekla-1980	Hekla/Hekla	1980	17/08/1980	145 km NNE of top crater	Plinian
Katla-1755	Mýrdalsjökull/Katla	1755	26/10/1997	~30 km E of caldera center	phreatomagmatic
Laki-SnV	Lakagígar/Grímsvötn	1783	15/08/1983	Eystrisker ~2 km NW of Hverfjall 1 vent	phreatomagmatic
Laki-SnVII	Lakagígar/Grímsvötn	1783	18/08/1983	Rootless cone tephra	phreatomagmatic
Orae-s13	Öræfajökull/Öræfajökull	1362	18/08/1993	11 km SSE of top crater	Plinian
Orae-s1	Öræfajökull/Öræfajökull	1362	18/08/1993	11 km SSE of top crater	Plinian or surge deposit
Reyk-1227	Karl/Yngri Stampar/Reykjanes	1227	15/03/1984	--	phreatomagmatic
Snae-1	Snæfellsjökull	~200	20/09/1992	3.5 km E of top crater	Plinian
Veid-1477	Veðivötn fissure/Bárðarbunga	1477	10/07/1976	~200 km NE of source	phreatomagmatic

## Methods

**Fig. 2:**
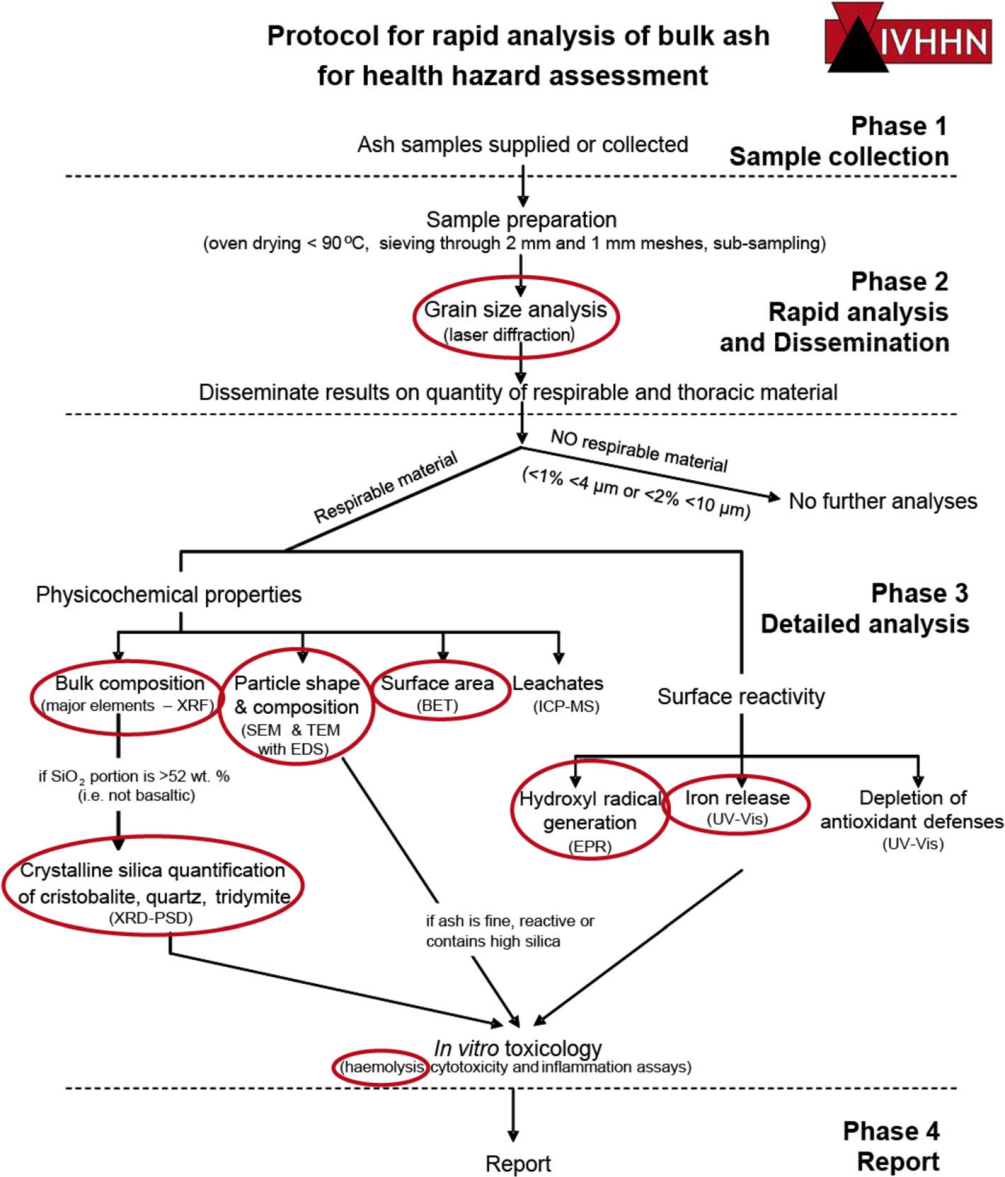
IVHHN rapid-response protocol used to assess the health hazard posed by volcanic ash. Circled analyses were performed for this study. No leachate analyses or cell-based toxicity experiments were conducted as samples were not collected fresh. Protocol adapted from Damby et al. [15]

The employed IVHHN protocol (Fig. 2), explained in detail previously [8, 15, 16], was designed for rapid assessment and uses the < 1 mm ash fraction rather than a respirable fraction due to the quantity of ash required for analyses and timeframe for separating sufficient fine ash for the range of analyses. While other health-focused studies of volcanic ash use a respirable isolate, particularly in toxicology studies [41–43], use of the < 1 mm fraction for all analyses here is considered appropriate as the parameters of interest for physicochemical characterization generally correspond between the bulk material and respirable fraction [44]. All samples were dried in an oven at 80 °C for 12 h and then sieved (Endecotts woven wire stainless steel sieves) first through a 2 mm and then subsequently a 1 mm sieve. No impact on particle characteristics is expected from this preparation; however, if leachable elements are of concern, a sample split should be taken prior to drying and sieving as outlined elsewhere for best practice [45, 46].

Physicochemical assessment was conducted on all samples except where sample quantities were insufficient or it was believed that the samples were unrepresentative of the original ash deposit. Samples for analysis by transmission electron microscopy (TEM) were chosen based on results of particle size analyses, where samples Hekla-4 and Laki-SnVII contained the highest abundance of < 1 μm material (see section 3.1). Samples selected for surface area analyses are the finest grained (most abundant < 4 μm fraction) that also represent the breadth of magmatic compositions and the range of volcanoes considered. Table 2 provides a summary of the analyses conducted on each sample. No evidence of alteration was noted during physicochemical characterization of the archived ash samples, but full toxicological testing was not pursued because the samples were, in some cases, several thousand years old and, inevitably, will have experienced surface weathering prior to collection, thereby affecting their toxicity profile [47].

**Table 2:** Summary of experiments conducted on volcanic ash samples

Sample	PSD	XRF	XRD	SEM	TEM	SSA	EPR	Fe	Hem
Askja-55d	x	x		x					
Askja-56a	x	x		x					
Askja-59b	x	x		x		x	x	x	x
Hekla-4	x	x	x	x	x	x	x	x	x
Hekla-1158		x							
Hekla-1980	x	x	x	x		x	x	x	x
Katla-1755	x	x	x	x		x	x	x	x
Reyk-1227	x	x	x	x					
Laki-SnV	x	x	x						
Laki-SnVII	x	x		x	x	x	x	x	x
Orae-s13	x	x	x	x					
Orae-s1	x	x	x	x		x	x	x	x
Snae-1			x	x					
Veid-1477	x	x	x	x					

Insufficient sample mass limited the range of analyses performed on some samples. Analyses include: particle size distribution (PSD); X-ray fluorescence (XRF); X-ray diffraction (XRD); scanning electron microscopy (SEM); transmission electron microscopy (TEM); BET-N_2_ specific surface area (SSA); electron paramagnetic resonance (EPR); available surface iron (Fe); and particle-induced hemolysis (Hem)

### Bulk chemical composition

The chemical composition of ash samples was determined by X-ray fluorescence (XRF) using a PANalytical Axios Advanced XRF spectrometer at the Department of Geology, University of Leicester, UK. Major elements were analyzed on fused glass beads prepared from ignited powders, using 100% Li tetraborate flux with a sample to flux ratio 1:10. Data have been recalculated to include loss on ignition.

### Particle size analysis

Particle size data were collected by laser diffraction using a Malvern Mastersizer 2000 with Hydro MU attachment at the Department of Geography, University of Cambridge, UK. Ultrasonics were used to disaggregate samples and the results are from an average of three runs. Samples were measured with a refractive index appropriate for their chemical composition (after [48]) and an absorption coefficient of 0.1. Data were interpolated to include the 1–2 mm particles according to weight fractions of the sieved material in order to provide particle size data relative to the ‘ash’ (< 2 mm) fraction.

### Particle morphology

Imaging of volcanic ash by scanning electron microscopy (SEM) was carried out on a Hitachi SU-70 FEG SEM in the GJ Russell Microscopy Facility, Department of Physics, Durham University, UK. Samples were prepared for imaging by sprinkling ash onto a polycarbonate disc adhered to an aluminum SEM stub using a carbon sticky pad and coated with ~30 nm of carbon.

TEM was used to observe the morphology, chemistry, and structure of the nano-fraction in volcanic ash with a specific emphasis on respirable fibers. Analyses were carried out on a JEOL JEM-2100F FEG TEM at 200 kV in the GJ Russell Microscopy Facility, Department of Physics, Durham University, UK. Bulk ash samples were analyzed on holey carbon copper TEM grids.

### Crystalline silica identification

Powder X-ray diffraction (XRD) was used to identify crystalline silica phases in the ash. Crystalline silica polymorphs have been singled out for identification (and quantification when present) because they are classed as human carcinogens [49] and may cause the fibrotic lung disease silicosis [50]. Sub-samples of ash were prepared as a thin smear on a silicon zero diffraction plate and data collected using a Bruker Analytical D8 ADVANCE diffractometer with DAVINCI design in the Department of Chemistry, Durham University, UK.

### Particle specific surface area

Specific surface area (SSA) corresponds strongly with particle toxicity as it is a measure of the maximum available surface on which reactions can occur. We used the BET (Brunauer–Emmett-Teller) method of specific surface area analysis using nitrogen adsorption with a Micromeritics TriStar 3000 Surface Area and Porosimetry Analyser in the Department of Chemistry, Durham University, UK. Prior to analysis, samples were degassed under nitrogen at 110 °C for a minimum of 2 h.

### Free radical generation

The surface reactivity of particles was assessed by their ability to generate free radicals, which are known to be potential inflammatory and carcinogenic factors [51, 52]. Electron Paramagnetic Resonance (EPR) spectroscopy was used to quantify the generation of hydroxyl radicals (HO^•^) using the spin-trapping technique through replication of the Fenton reaction [53]. Measurements were taken at 30 min using a Miniscope 100 ESR spectrometer, Magnettech at the Università degli Studi di Torino, Italy. The integrated amplitude of the peaks generated is proportional to the number of radicals generated. Data are the average of three runs and are expressed per unit surface area (see section 2.4). Three previously analyzed samples of ash from other volcanoes were included for comparison (see [54]).

Given that some samples used in this study are ancient, EPR experiments were conducted on samples as they were collected (un-ground) as well as after light grinding. Grinding exposes fresh surfaces, which provides an indication of the reactivity expected for non-weathered samples [55]. Approximately 500 mg of ash was ground in a Retsch MM2 mixer mill at a frequency of 27 Hz for 60 min. Samples were analyzed immediately after grinding. The specific surface area of samples was only determined for the un-ground samples as insufficient material was available to also analyze the ground samples, but previous experience has shown that the change in SSA is minimal as grinding abrades ash surfaces with little fragmentation of particles [55].

### Surface iron release

Experiments were conducted to determine the amount of surface iron available for participation in the iron-based reactivity assessed by EPR. Following previously detailed methods [54], ash was incubated at 37 °C with the Fe^2+^-specific chelator ferrozine with and without ascorbic acid to measure total Fe and Fe^2+^, respectively (Fe^3+^ was calculated by difference). Measurements were taken every 24 h for 7 days in a Uvikon spectrophotometer (562 nm) at the Università degli Studi di Torino, Italy, a time-point previously identified as sufficient to approximate maximum iron removal [54]. Three previously analyzed samples (from other volcanoes, as above) were included for comparison. Results are expressed per unit surface area and are the average of two separate experiments. Only un-ground samples were analyzed due to the limited amount of ash available.

### Hemolysis assay

The toxicity of the ash was assessed with the hemolysis (red blood cell lysis) assay. A positive result signifies the ability of a sample to rupture a biological membrane, which is an indicator for the potential to cause cellular damage. For crystalline silica, a direct relationship between hemolysis and disease has been reported [56], thereby serving as one end point related to inflammation to be tested. Human red blood cells were exposed to a range of ash concentrations in saline from 0.31 to 1 mg ml^−1^ for 30 mins at the University of Edinburgh, UK following methods previously detailed [44]. DQ12 quartz was used as a positive control and TiO_2_ was used as a negative control. Data are reported as a percentage of complete cellular lysis as determined with Triton X-100 (0.1%). Samples were not ground prior to analysis. Analyses were carried out on bulk material, which is common practice for volcanic ash [8, 15, 57], as hemolysis on bulk and respirable samples have shown similar results [44].

## Results

### Physicochemical characterization

**Fig. 3:**
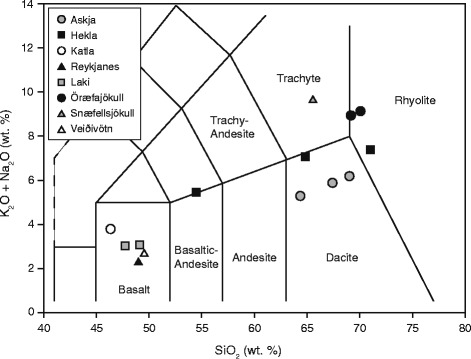
Bulk compositions of volcanic ash samples. Sample compositions are plotted according to magma type as total alkali (K_2_O + Na_2_O) versus silica (SiO_2_). Data for Snæfellsjökull sample (Snae-1) from Martin and Sigmarsson [77]

The samples selected for this study are from historical and prehistoric eruptions of Icelandic volcanoes, and span the range of magma compositions (from basaltic to rhyolitic) that could be expected from Icelandic volcanism (Fig. 3). All samples were generally coarse grained (Table 3), with the exception of Laki-SnVII, an ash sample from the 1783–84 Laki eruption, and Hekla-4, erupted c.a. 4.2 ka B.P, which contained abundant respirable material (5.1 and 11.3 vol.% < 4 μm, respectively). Particle sizes observed by SEM imaging qualitatively corroborated laser diffraction data. Samples Snae-1 and Hekla-1158 were not analyzed for particle size since they were too coarse-grained.

**Table 3:** Particle size and specific surface area results for select volcanic ash samples

Composition	Sample	< 1 μm	< 2.5 μm	< 4 μm	< 10 μm	< 100 μm	SSA
Basalt	Katla-1755	0.05	0.55	0.99	2.63	33.00	0.41
	Laki-SnVII	0.65	3.00	5.07	11.40	60.74	1.00
	Laki-SnV	0.35	1.22	1.90	4.72	54.12	
	Reyk-1227	0.00	0.13	0.27	1.13	27.32	
	Veid-1477	0.00	0.00	0.00	0.00	70.44	
Basaltic-Andesite	Hekla-1980	0.00	0.27	0.54	1.80	55.87	0.45
Dacite	Askja-59b	0.21	1.38	2.59	7.39	54.97	0.60
	Askja-55d	0.17	1.28	2.47	7.36	46.05	
	Askja-56a	0.21	1.39	2.64	7.85	49.93	
Rhyolite	Orae-s1	0.39	2.12	3.86	10.51	62.03	0.90
	Orae-s13	0.26	1.51	2.74	7.66	55.61	
	Hekla-4	1.28	6.59	11.26	26.91	77.01	1.64

Particle size data are presented as cumulative volume % (of 2 mm) for health-relevant fractions. Specific surface area (SSA) data are m^2^ g^−1^. Samples are ordered according to increasing SiO_2_ content. All data are averages of three discrete analyses

**Fig. 4:**
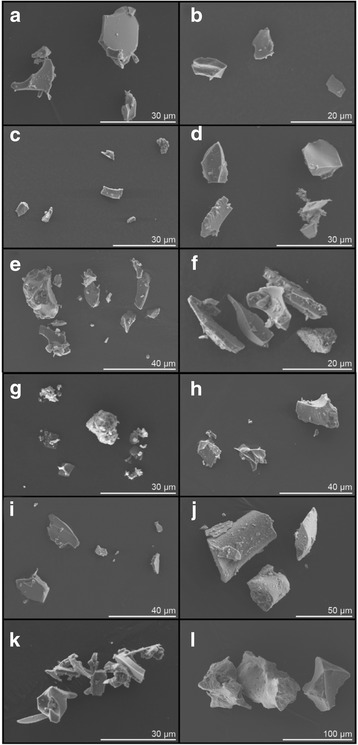
Selected scanning electron micrographs of volcanic ash samples. (**a**) Askja-55d, (**b**) Askja-56a, (**c**) Askja-59b, (**d**) Hekla-1980, (**e**) Hekla-4, (**f**) Katla-1755, (**g**) Laki-SnVII, (**h**) Orae-s1, (**i**) Orae-s13, (j) Reyk-1227, (k) Snae-1 and (l) Veid-1477. All images were collected at 8.0 kV and ~14 mm working distance

Ash particles of all sizes (< 1 mm) are generally angular with fractured surfaces (see Fig. 4), as expected for volcanic ash [8, 15–17]. All samples were visually similar except for Laki-SnVII, which was sampled from two ashfall units produced by rootless eruptions that took place as the basaltic lava advanced over wetlands [58]. This sample contained aggregates of fine material and fine particles adhered to larger particles (Fig. 4g), and also contained diatom frustules (which are not discussed further here but have been identified in deposits from other eruptions [59]).

**Fig. 5:**
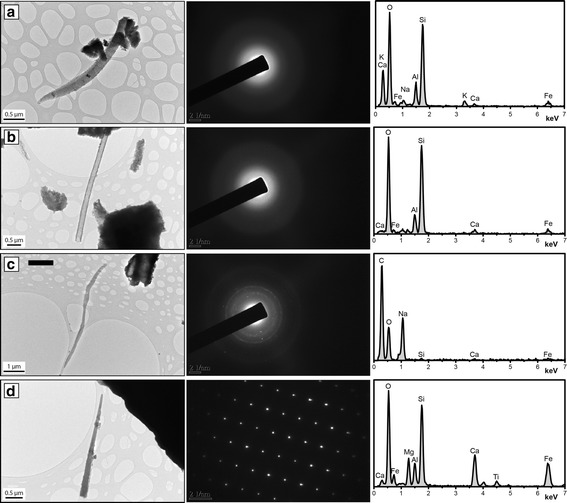
Transmission electron microscope analysis of respirable material in select volcanic ash samples. TEM data of fiber-like particles in Hekla-4 (**a**) and Laki-SnVII (**b**-**d**) with corresponding selected area diffraction patterns and chemical (EDS) analyses (middle and right columns of panels, respectively): (**a**) completely amorphous fiber dominated by oxygen, silicon, aluminum, potassium, calcium and iron; (**b**) completely amorphous fiber consisting of predominantly silicon and oxygen with lesser amounts of aluminum, iron and calcium; (**c**) semi-crystalline fiber consisting of carbon, sodium and oxygen; (**d**) well-crystalline fiber predominantly comprised of silicon and oxygen with calcium, iron and magnesium, and lesser amounts of titanium

Fibers were rare, but present, in both samples investigated by TEM (Fig. 5). Three types of fiber-like particles were identified in Laki-SnVII: completely amorphous (Fig. 5b), semi-crystalline comprised of sodium and oxygen (Fig. 5c), and well-crystalline comprised of silicon and oxygen with lesser amounts calcium, iron, magnesium, aluminum, and titanium (Fig. 5d). Although the actual mineral was not identifiable, the well-crystalline fibers are not related to asbestiform minerals or fibrous zeolites based on database indexing. The three different types of fibers in Laki-SnVII were encountered with equal frequency. In Hekla-4, only amorphous, fiber-like particles were identified (Fig. 5a).

The specific surface areas measured range from 0.4 to 1.6 m^2^ g^−1^ (Table 3). Hekla-4 had the largest surface area of all samples analyzed. These surface area values are in keeping with expected values from previous analyses of ash samples [8, 15–17].

Almost all samples analyzed lacked a detectable crystalline silica component, being predominantly comprised of glass and some feldspar. The exception was Snae-1, which contained detectable amounts of quartz. There was insufficient mass of this sample for quantitative analysis, however. No sample contained cristobalite or tridymite.

### Particle surface reactivity and toxicity

**Fig. 6:**
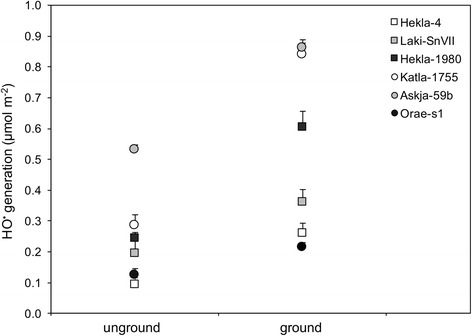
Comparison of surface reactivity for ground and un-ground volcanic ash samples. Production of hydroxyl radicals (HO^•^) was measured by spin-trapping and electron paramagnetic resonance after 30 min through replication of the iron-catalyzed Fenton reaction. Grinding was intended to restore the sample surface in order to represent HO^•^ production from freshly collected samples. Data are presented per unit surface area. Error bars represent standard error (*n* = 3)

**Fig. 7:**
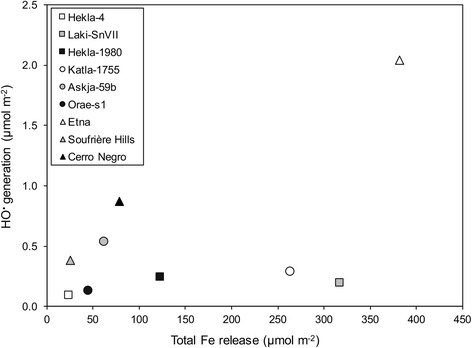
Comparison of hydroxyl radical production with removable surface iron for select volcanic ash samples. Hydroxyl radicals (HO^•^) produced through replication of the iron-catalyzed Fenton reaction by unground samples were determined by spin-trapping and electron paramagnetic resonance after 30 min. The total amount of available iron extracted from unground samples by chelation is presented for a time-point of 7 days. Hydroxyl radical generation and iron release are both expressed per unit surface area. The three comparative samples have been used previously [54] and were reanalyzed for this study: Mt. Etna, Sicily (2002, basaltic), Soufrière Hills, Montserrat (5/6/99, andesitic), Cerro Negro, Nicaragua (1995, basaltic)

All samples were able to generate hydroxyl radicals (Fig. 6), even if the amount of radicals was low when compared with standard ash samples analyzed concurrently (see also Fig. 7). Moreover, all samples generated more hydroxyl radicals when ground than when analyzed as collected (Fig. 6). That ‘refreshing’ the sample surfaces resulted in increased radical generation is consistent with previous data [54], as weathered ash samples are more oxidized and less reactive than freshly collected samples [55].

The use of previously analyzed andesitic and basaltic samples provides benchmarks for the compositional range of the current samples. As expected from the spectrum of samples analyzed, there is a range in the amount of chelatable iron, with basaltic samples (e.g., Katla-1755 and Laki-SnVII) releasing more iron than dacitic and rhyolitic samples (e.g., Hekla-4, Orae-s1, Askja-59b). However, there is no correlation between the amount of iron mobilized from the surface of un-ground particles and iron-catalyzed hydroxyl radical generation (Fig. 7). Askja-59b, a dacitic sample, generated the most hydroxyl radicals.

**Fig. 8:**
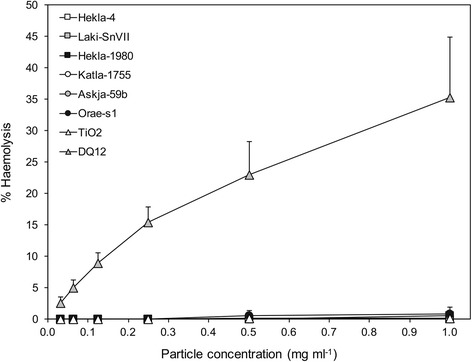
Hemolytic potential of selected volcanic ash samples. Dose-dependent hemolysis as a percentage of complete cell lysis (0.1% Triton X-100) for human red blood cells treated with 0.31 to 1 mg ml^−1^ sample concentrations of volcanic ash. TiO_2_ and DQ12 (quartz) are negative and positive particle controls, respectively. Error bars are the standard error of the mean (*n* = 3)

None of the samples were found to be hemolytic compared to the negative particle control, and were far less reactive than the positive quartz control (Fig. 8).

## Discussion

A suite of analyses was conducted in accordance with the IVHHN protocol (Fig. 2) to constrain the potential respiratory health hazard posed in the event of future eruptions of Iceland’s volcanoes in order to inform pre-eruption mitigation policy and preliminary risk assessments whilst syn-eruption data are being collected. The samples selected for this study, from historical and prehistoric eruptions of Icelandic volcanoes, encompass the full range of magma compositions that could be expected from Icelandic volcanism (Fig. 3). Studies on the recent eruptions of Eyjafjallajökull and Grímsvötn [8] were also conducted following the IVHHN protocol and are considered alongside the present study.

### Respirability of Icelandic volcanic ash

Determining the potential for volcanic ash to be inhaled is of utmost importance when assessing the respiratory hazard. Particle size distributions of ash samples are a function of the eruption intensity (which differs over 3 to 4 orders of magnitude among individual events considered here) as well as size fractionation during transport to the site of deposition. Therefore, exposures are temporally and spatially variable throughout and following an eruption. Only 1 to 3 samples were sourced per eruption in the present study and, by and large, samples were collected at discrete locations relatively close to source; as such, the particle size measurements are considered an indication of potential rather than being representative of the finest tephra produced.

In general, intermediate to silicic eruptions result in finer particle size distributions than basaltic eruptions [48]; aside from the Laki samples, the data here are in keeping with this trend (Table 3). However, basaltic eruptions in Iceland have also produced fine-grained ash, such as previous eruptions of Katla, with ash reaching mainland Europe [60]. The notable example in the present study is Laki-SnVII (5.07 vol.% < 4 μm). While these data are not likely representative of the cumulative ashfall of the eruption, as evidenced by the difference in respirable material between Laki-SnVII and Laki-SnV (1.90 vol.% < 4 μm), they highlight the potential for discrete events during an eruption to pose a variable respiratory hazard. This was also observed for the eruption of Eyjafjallajökull, with the abundance of fine material differing amongst the four phases of the eruption [1, 8].

Ash particles were angular and blocky, which is consistent with previous studies, and is also observed for respirable ash [41]. Experimentally, fine-grained ash of this morphology effectively deposits in the alveolar and tracheobronchial regions of the lungs [18], which should be expected for future exposures. To date, including the current study, the presence and relative abundance of fiber-like particles in ash has been exceptionally rare by number and is sample specific (samples from Hekla and Laki analysed here and also see reference [8]). We note, however, that fibers were identified by TEM in both samples analyzed here and suggest that such analyses be conducted during future eruptions as the general occurrence of nano-fibers in ash is poorly constrained to date. Even still, no fibrous minerals with established toxicity profiles have been identified, thereby limiting concerns for future exposures.

The experiences of the 2010 Eyjafjallajökull and 2011 Grímsvötn eruptions, the extensive evidence of ashfall reaching UK/Europe from previous eruptions in Iceland (e.g., [61]), and the quantification of respirable material in samples from the historical eruptions studied herein demonstrate the potential for long-range transport of inhalable ash from eruptions spanning the compositional range of Iceland’s volcanoes. Even ‘weak’ eruptions and low-altitude plumes can transport ash to distances of >1000 km [1, 10]. Existing tephra dispersion models can accurately capture the timing of arrival of an ash cloud [62], but are unable to reliably predict the abundance of fine ash or ground-level mass concentrations. In the event that dispersion models predict that ash will reach the UK/Europe from an Icelandic source, existing ground-based air quality networks, such as those used to monitor compliance with the EU Ambient Air Quality Directive, and syndromic surveillance systems can be used to monitor exposure and community health during the incident. Particular attention should be paid to areas with existing PM burdens, as small increases in PM concentrations can lead to an increase in exceedance days, and combined exposure to ash and anthropogenic pollution can result in a heightened immune response in vitro [42].

### Crystalline silica hazard of Icelandic ash

The minerals of primary concern in volcanic ash are the crystalline silica polymorphs due to their potential to cause fibrotic lung disease and cancer [49]. It is presently unclear whether volcanic crystalline silica can cause disease [41, 63, 64], but volcanic ash has been shown to activate the same biological platform involved in the development of some other particle-induced lung diseases, including silicosis [65]. Until this is further clarified, all rapid health assessments consider the amount of crystalline silica present.

The crystalline silica hazard posed was low for all samples analyzed. This is consistent with previous studies on silicic volcanic products in Iceland, where quartz is exceedingly rare as a phenocryst phase [66], as well as the results from the previous IVHHN and other studies on Eyjafjallajökull and Grímsvötn ash, which found minor quartz in select Eyjafjallajökull samples (< 3 wt.%) and no detectable crystalline silica in Grímsvötn tephra [8, 18, 67]. The exception in the present study was Snae-1 (glass compositions of 66–69 wt.% SiO_2_ [68]); however, the bulk sample was exceptionally coarse grained (91 wt.% > 2 mm material), so was considered non-respirable and was not analyzed further.

Although cristobalite and tridymite were not present in the samples analyzed, these phases are able to form in lava flows and domes from volcanoes with basaltic-andesite to rhyolitic compositions that have undergone post-emplacement mineralization or alteration (see review in [44]), and can be present in substantial quantities through spherulitic growth in dome lavas in Iceland [69]. Critically, the presence of crystalline silica is not ubiquitous throughout an eruption, as evidenced by the 2008 eruption of Chaitén volcano, where substantial cristobalite was only detected after dome growth (and collapse) began [70]. Therefore, these phases may be present if future eruptions incorporate older material, or in the event of new dome growth during an eruption. Given that no ash was available from historical eruptions involving disruption of a dome, preliminary SEM and XRD analyses were conducted on dome samples from Torfajökull and Krafla. Crystalline silica was present as both cristobalite and tridymite; however, consideration of the resultant abundance of crystalline silica in ash from an eruption of either location would be highly speculative and is not further discussed. Accordingly, we suggest case-by-case consideration of crystalline silica in the ash from future intermediate to silicic eruptions, despite its absence in the present study, as it is considered a hazard that can evolve throughout an eruption.

### Potential toxicity of Icelandic ash

The inherent variability of volcanic ash across eruptions has prohibited consensus on when (or if) ash will be pathogenic. To date, the IVHHN approach has been to screen samples using the employed tests for free radical production and membrane damage (hemolysis) as the pathways through which volcanic ash can initiate a pro-inflammatory response are largely unconstrained [8, 65]. While other biological tests could be employed, the chosen analyses are low-cost and rapid, making them ideal for a screening approach, and account for the two predominant concerns: crystalline silica and reactive surface iron [54, 71]. Together with physicochemical profiling of ash, this allows us to assess the value of pursuing additional, costlier immune cell-based experiments since these tests are not definitive indicators of toxicity alone [72], regardless of the inherent variability of volcanic ash. The two endpoints considered were unable to offer predictive insight into the potential variability in toxicity of ash from future eruptions of Iceland’s volcanoes, but, as mentioned, no further toxicology work was conducted as these samples were not collected fresh and, accordingly, results would have limited applicability [47].

The propensity to generate free radicals serves as one candidate mechanism through which some samples of volcanic ash (including Icelandic ash) can initiate a pro-inflammatory response in vitro [8, 41, 73]. Work on volcanic ash frequently focuses on iron-catalyzed free radical generation [54, 55, 74], which can become prolonged when iron is present in a specific oxidative and coordinative state on the surface of particulate matter [75]. While a dependence on magmatic composition has been observed previously [54, 55], where Fe-rich mafic samples generate more hydroxyl radicals than comparatively Fe-poor silicic samples, a strong trend was not observed in the present study. Mafic samples Katla-1755, Hekla-1980 and Laki-SnVII were only slightly more reactive than silicic samples Orae-s1 and Hekla-4. Askja-59b generated the greatest number of hydroxyl radicals of the test samples despite having a dacitic composition and, therefore, a lower abundance of (surface) iron than basaltic Katla-1755, Hekla-1980 and Laki-SnVII (Figs. 6 and 7). The compositionally aberrant production of radicals by Askja-59b has been observed previously for felsic ash (dacitic Pinatubo sample in reference [54]), albeit infrequently, emphasizing that high-silica/low-iron compositions do not necessarily display lower reactivity than low-silica/high-iron compositions. This is likely because different phases in ash are variably responsible for radical production [54], and hydroxyl radical generation is governed by the coordination and redox state of discrete sites on the particle surface [75]. Accordingly, fresh samples should continue to be screened in this manner at the time of an eruption as post-eruptive processing of particles in the eruptive plume, atmosphere and environment, in addition to inherent variability, can affect particle surfaces to variable extents.

Despite the lack of hemolysis observed here and for ash from other volcanoes [8, 15], hemolysis retains value as a screening tool. Recent experimental work has shown that crystalline silica-bearing ash can activate the NLRP3 inflammasome [65], a mechanism central to the initiation of crystalline silica-induced disease. For reactive surfaces like quartz, the property of being hemolytic carries with it the property of activating the NLRP3 inflammasome [76], thereby linking hemolytic activity with inflammogenic activity. This discrepancy between negative hemolysis results and the evidence for ash-induced inflammation in vitro has yet to be reconciled, particularly for ash containing appreciable crystalline silica, and is the focus of on-going toxicology work.

## Conclusions

Prior to the recent eruptions of Eyjafjallajökull and Grímsvötn, little was known about the toxicity of ash, or the variability thereof, from Iceland’s volcanoes. Studies conducted in Iceland in the aftermath of Eyjafjallajökull, in particular, reported immediate symptoms, both near source and at distances greater than 100 km (Reykjavík), as well as long-term symptoms in communities with chronic exposure to ash. Transient increases in PM were observed in the UK and Europe due to long-range transport of ash from both eruptions, but there was no risk of chronic exposure from resuspended material and no conclusive evidence for a resultant decrease in population health. The events prompted the need to evaluate the hazard posed by future eruptions, however, and a particle-screening approach was adopted for the present study to constrain the range of toxicity-relevant particle characteristics that may result from future eruptions.

The samples analyzed here span the compositional range of Iceland’s volcanoes (basaltic to rhyolitic) and were mostly from eruptions that can be grouped as moderate to large in terms of eruption intensity and erupted volume (0.1 km^3^ to 10 km^3^). Specific samples from the various eruptions contained substantial respirable material, and we should expect that future explosive eruptions of Iceland’s volcanoes across the compositional spectrum could produce respirable ash, which may augment ambient PM levels considerably. We expect future Icelandic eruptions to generate ash which has low crystalline silica content, unless an existing dome is disrupted, to have a low surface area and to have insubstantial fiber-like particles. Nevertheless, if inhalable in size, the ash may be reactive in vivo. That ‘refreshing’ the sample surfaces resulted in increased radical generation emphasizes that analysis of fresh ash (whenever possible) is prudent for determining the immediate hazard to public health. The ranges defined for the health-relevant physicochemical properties in this detailed characterization can be used as a qualitative evidence base for future hazard and risk assessment and for scenario-building purposes, but we suggest that eruption-specific monitoring of ash continue to be undertaken as ash characteristics vary as eruptions evolve. Additionally, we emphasize that gas and soluble-species hazards are not addressed in the present study and warrant incorporation into population health assessments during future eruptions.

## Abbreviations

BET: Brunauer–Emmett-Teller
EDS: Energy-dispersive X-ray spectroscopy
EPR: Electron paramagnetic resonance spectroscopy
HPA: Health Protection Agency
IVHHN: International Volcanic Health Hazard Network
PM: Particulate matter
PSD: Particle size distribution
SEM: Scanning electron microscopy
SSA: Specific surface area
TEM: Transmission electron microscopy
XRD: X-ray diffraction
XRF: X-ray fluorescence

## References

[1] Gudmundsson MT, Thordarson T, Höskuldsson Á, Larsen G, Björnsson H, Prata FJ, Oddsson B, Magnússon E, Högnadóttir T, Petersen GN (2012). Ash generation and distribution from the April-May 2010 eruption of Eyjafjallajökull, Iceland. Sci Rep..

[2] Gudmundsson G (2011). Respiratory health effects of volcanic ash with special reference to Iceland. A review. Clin Respir J.

[3] Carlsen HK, Hauksdottir A, Valdimarsdottir UA, Gíslason T, Einarsdottir G, Runolfsson H, Briem H, Finnbjornsdottir RG, Gudmundsson S, Kolbeinsson TB (2012). Health effects following the Eyjafjallajökull volcanic eruption: a cohort study. BMJ.

[4] Seaton A, Godden D, MacNee W, Donaldson K (1995). Particulate air pollution and acute health effects. Lancet.

[5] World Health Organization (2013). Review of evidence on health aspects of air pollution – REVIHAAP project: final technical report.

[6] Loomis D, Grosse Y, Lauby-Secretan B, El Ghissassi F, Bouvard V, Benbrahim-Tallaa L, Guha N, Baan R, Mattock H, Straif K (2013). The carcinogenicity of outdoor air pollution. Lancet Oncol.

[7] Elliot AJ, Singh N, Loveridge P, Harcourt S, Smith S, Pnaiser R, Kavanagh K, Robertson C, Ramsay CN, McMenamin J (2010). Syndromic surveillance to assess the potential public health impact of the Icelandic volcanic ash plume across the United Kingdom, April 2010. Eur Secur.

[8] Horwell CJ, Baxter PJ, Hillman SE, Calkins JA, Damby DE, Delmelle P, Donaldson K, Dunster C, Fubini B, Hoskuldsson A (2013). Physicochemical and toxicological profiling of ash from the 2010 and 2011 eruptions of Eyjafjallajökull and Grímsvötn volcanoes, Iceland using a rapid respiratory hazard assessment protocol. Environ Res.

[9] Brand H, Krafft T (2010). The Icelandic ash cloud and other erupting health threats: what role for syndromic surveillance?. Eur J Pub Health.

[10] Stevenson JA, Loughlin SC, Font A, Fuller GW, MacLeod A, Oliver IW, Jackson B, Horwell CJ, Thordarson T, Dawson I (2013). UK monitoring and deposition of tephra from the May 2011 eruption of Grímsvötn, Iceland. J Appl Volcanol..

[11] Tesche M, Glantz P, Johansson C, Norman M, Hiebsch A, Ansmann A, Althausen D, Engelmann R, Seifert P (2012). Volcanic ash over Scandinavia originating from the Grímsvötn eruptions in May 2011. J Geophys Res.

[12] Harrison RM, Yin J (2000). Particulate matter in the atmosphere: which particle properties are important for its effects on health?. Sci Total Environ.

[13] Oberdörster G, Maynard A, Donaldson K, Castranova V, Fitzpatrick J, Ausman K, Carter J, Karn B, Kreyling W, Lai D (2005). Principles for characterizing the potential human health effects from exposure to nanomaterials: elements of a screening strategy. Part Fibre Toxicol..

[14] Horwell CJ, Baxter PJ, Hillman SE, Damby DE, Delmelle P, Donaldson K, Dunster C, Calkins JA, Fubini B, Hoskuldsson A, et al. Respiratory health hazard assessment of ash from the 2010 eruption of Eyjafjallajökull volcano, Iceland. A summary of initial findings from a multi-centre laboratory study. Int Volcanic Health Hazard Net Rep. 2010:1-10.

[15] Damby DE, Horwell CJ, Baxter PJ, Delmelle P, Donaldson K, Dunster C, Fubini B, Murphy FA, Nattrass C, Sweeney S (2013). The respiratory health hazard of tephra from the 2010 Centennial eruption of Merapi with implications for occupational mining of deposits. J Volcanol Geotherm Res.

[16] Le Blond JS, Horwell CJ, Baxter PJ, Michnowicz SAK, Tomatis M, Fubini B, Delmelle P, Dunster C, Patia H (2010). Mineralogical analyses and in vitro screening tests for the rapid evaluation of the health hazard of volcanic ash at Rabaul volcano, Papua New Guinea. Bull Volcanol.

[17] Horwell CJ, Stannett GW, Andronico D, Bertagnini A, Fenoglio I, Fubini B, Le Blond JS, Williamson BJ (2010). A physico-chemical assessment of the health hazard of Mt. Vesuvius volcanic ash. J Volcanol Geotherm Res.

[18] Lähde A, Gudmundsdottir SS, Joutsensaari J, Tapper U, Ruusunen J, Ihalainen M, Karhunen T, Torvela T, Jokiniemi J, Järvinen K (2013). In vitro evaluation of pulmonary deposition of airborne volcanic ash. Atmos Environ.

[19] Arnalds O, Thorarinsdottir E, Thorsson J, Waldhauserova P, Agustsdottir A (2013). An extreme wind erosion event of the fresh Eyjafjallajokull 2010 volcanic ash. Sci Rep..

[20] Thorsteinsson T, Jóhannsson T, Stohl A, Kristiansen N. High levels of particulate matter in Iceland due to direct ash emissions by the Eyjafjallajökull eruption and resuspension of deposited ash. J Geophys Res. 2012;117(B9):B00C05.

[21] Carlsen HK, Gislason T, Benediktsdottir B, Kolbeinsson TB, Hauksdottir A, Thorsteinsson T, Briem H (2012). A survey of early health effects of the Eyjafjallajökull 2010 eruption in Iceland: a population-based study. BMJ.

[22] Carlsen HK, Gislason T, Forsberg B, Meister K, Thorsteinsson T, Jóhannsson T, Finnbjornsdottir R, Oudin A (2015). Emergency Hospital Visits in Association with Volcanic Ash, Dust Storms and Other Sources of Ambient Particles: A Time-Series Study in Reykjavík, Iceland. Int J Environ Res Public Health.

[23] Hlodversdottir H, Petursdottir G, Carlsen HK, Gislason T, Hauksdottir A (2016). Long-term health effects of the Eyjafjallajökull volcanic eruption: a prospective cohort study in 2010 and 2013. BMJ Open.

[24] Horwell CJ, Baxter PJ (2006). The respiratory health hazards of volcanic ash: a review for volcanic risk mitigation. Bull Volcanol.

[25] Cadelis G, Tourres R, Molinie J, Petit RH (2013). Exacerbations d’asthme en Guadeloupe et éruption volcanique à Montserrat (70 km de la Guadeloupe). Rev Mal Respir.

[26] Newnham RM, Dirks KN, Samaranayake D (2010). An investigation into long-distance health impacts of the 1996 eruption of Mt Ruapehu, New Zealand. Atmos Environ.

[27] Oudin A, Carlsen HK, Forsberg B, Johansson C (2013). Volcanic Ash and Daily Mortality in Sweden after the Icelandic Volcano Eruption of May 2011. Int J Environ Res Public Health.

[28] Stevenson JA, Loughlin S, Rae C, Thordarson T, Milodowski AE, Gilbert JS, Harangi S, Lukács R, Højgaard B, Árting U, et al. Distal deposition of tephra from the Eyjafjallajökull 2010 summit eruption. J Geophys Res. 2012;117(B9):B00C10.

[29] Kar-Purkayastha I, Horwell CJ, Murray V (2012). Review of Evidence on the Potential Health Impacts of Volcanic Ash on the Population of the UK and ROI.

[30] Thordarson T, Höskuldsson Á (2008). Postglacial volcanism in Iceland. Jökull.

[31] Larsen G, Eiríksson J (2008). Holocene tephra archives and tephrochronology in Iceland—a brief overview. Jökull.

[32] Lawson IT, Swindles GT, Plunkett G, Greenberg D (2012). The spatial distribution of Holocene cryptotephras in north-west Europe since 7 ka: implications for understanding ash fall events from Icelandic eruptions. Quat Sci Rev.

[33] Larsen G, Gudmundsson MT, Björnsson H (1998). Eight centuries of periodic volcanism at the center of the Iceland hotspot revealed by glacier tephrostratigraphy. Geology.

[34] Schmidt A, Leadbetter S, Theys N, Carboni E, Witham CS, Stevenson JA, Birch CE, Thordarson T, Turnock S, Barsotti S (2015). Satellite detection, long-range transport, and air quality impacts of volcanic sulfur dioxide from the 2014–2015 flood lava eruption at Bárðarbunga (Iceland). J Geophys Res.

[35] Ilyinskaya E, Schmidt A, Mather TA, Pope FD, Witham C, Baxter P, Jóhannsson T, Pfeffer M, Barsotti S, Singh A, Sanderson P (2017). Understanding the environmental impacts of large fissure eruptions: Aerosol and gas emissions from the 2014–2015 Holuhraun eruption (Iceland). Earth Planet Sci Lett..

[36] Witham CS, Oppenheimer C, Horwell CJ (2005). Volcanic ash leachates: a review and recommendations for analysis methods. J Volcanol Geotherm Res.

[37] Jakobsson SP (1979). Outline of the petrology of Iceland. Jökull.

[38] Sæmundsson K (1979). Outline of the geology of Iceland. Jökull.

[39] Thordarson T, Larsen G (2007). Volcanism in Iceland in historical time: Volcano types, eruption styles and eruptive history. J Geodyn.

[40] Larsen G, Dugmore AJ, Newton AJ (1999). Geochemistry of historical-age silicic tephras in Iceland. Holocene.

[41] Damby DE, Murphy FA, Horwell CJ, Raftis J, Donaldson K (2016). The in vitro respiratory toxicity of cristobalite-bearing volcanic ash. Environ Res.

[42] Tomašek I, Horwell CJ, Damby DE, Barošová H, Geers C, Petri-Fink A, Rothen-Rutishauser B, Clift MJD (2016). Combined exposure of diesel exhaust particles and respirable Soufrière Hills volcanic ash causes a (pro-)inflammatory response in an in vitro multicellular epithelial tissue barrier model. Part Fibre Toxicol.

[43] Wilson M, Stone V, Cullen R, Searl A, Maynard R, Donaldson K (2000). In vitro toxicology of respirable Montserrat volcanic ash. Occup Environ Med.

[44] Damby DE. From Dome to Disease: The Respiratory Toxicity of Volcanic Cristobalite. *Durham University e-theses* 2012(Durham theses). 1–258.

[45] Stewart C, Horwell CJ, Plumlee G, Cronin S, Delmelle P, Baxter PJ, Calkins J, Damby DE, Morman S, Oppenheimer C. Protocol for analysis of volcanic ash samples for assessment of hazards from leachable elements. In: IAVCEI Commissions joint report: International Volcanic Health Hazard Network and Cities and Volcanoes. 2013. Protocol downloadable from: www.ivhhn.org.

[46] Ayris PM, Delmelle P, Pereira B, Maters EC, Damby DE, Durant AJ, Dingwell DB. Spatial analysis of Mount St. Helens tephra leachate compositions: implications for future sampling strategies. Bull Volcanol. 2015;77(7):60. 10.1007/s00445-015-0945-8 PMC4498446 26190880

[47] Horwell CJ, Damby DE, Hillier S. Respirable volcanic ash is distinct mineralogically, physicochemically and toxicologically from soils originating from weathered volcanic products. A comment on Cervini-Silva et al. (2014) “Lipid peroxidation and cytotoxicity induced by respirable volcanic ash”. J Hazard Mater. 2015(285):366–7. 10.1016/j.jhazmat.2014.10.059 25528235

[48] Horwell CJ (2007). Grain size analysis of volcanic ash for the rapid assessment of respiratory health hazard. J Environ Monitor.

[49] International Agency for Research on Cancer. Silica, some silicates, coal dust and para-aramid fibrils. In: IARC monographs on the evaluation of carcinogenic risk of chemicals to humans. Vol. 68. Lyon; 1997. p. 1–475. PMC5366849 9303953

[50] Leung CC, Yu ITS, Chen W (2012). Silicosis. Lancet..

[51] Hardy JA, Aust AE (1995). Iron in asbestos chemistry and carcinogenicity. Chem Rev.

[52] Kane AB, Kane AB, Boffetta P, Saracci R, Wilburn JD (1996). Mechanisms of Mineral Fibre Carcinogenesis. Mechanisms of Fibre Carcinogenesis.

[53] Fubini B, Mollo L, Giamello E (1995). Free radical generation at the solid/liquid interface in iron containing minerals. Free Rad Res.

[54] Horwell CJ, Fenoglio I, Fubini B (2007). Iron-induced hydroxyl radical generation from basaltic volcanic ash. Earth Planet Sci Lett.

[55] Horwell CJ, Fenoglio I, Ragnarsdottir KV, Sparks RSJ, Fubini B (2003). Surface reactivity of volcanic ash from the eruption of Soufrière Hills volcano, Montserrat, West Indies with implications for health hazards. Environ Res.

[56] Pavan C, Fubini B (2017). Unveiling the Variability of “Quartz Hazard” in Light of Recent Toxicological Findings. Chem Res Toxicol.

[57] Hillman SE, Horwell CJ, Densmore AL, Damby DE, Fubini B, Ishimine Y, Tomatis M (2012). Sakurajima volcano: a physico-chemical study of the health consequences of long-term exposure to volcanic ash. Bull Volcanol.

[58] Thordarson T, Self S (1993). The Laki (Skaftár Fires) and Grímsvötn eruptions in 1783-1785. Bull Volcanol.

[59] Van Eaton AR, Harper MA, Wilson CJN. High-flying diatoms: Widespread dispersal of microorganisms in an explosive volcanic eruption. Geology. 2013;41(11):1187–90.

[60] Larsen G. Katla: Tephrochronology and Eruption History. Dev Quat Sci. 2010;13:23–49.

[61] Watson EJ, Swindles GT, Savov IP, Lawson IT, Connor CB, Wilson JA (2017). Estimating the frequency of volcanic ash clouds over northern Europe. Earth Planet Sci Lett.

[62] Wilkins KL, Western LM, Watson IM. Simulating atmospheric transport of the 2011 Grímsvötn ash cloud using a data insertion update scheme. Atmos Environ. 2016(141):48–59.

[63] Horwell CJ, Williamson BJ, Donaldson K, Le Blond JS, Damby DE, Bowen L. The structure of volcanic cristobalite in relation to its toxicity; relevance for the variable crystalline silica hazard. Part Fibre Toxicol. 2012;9:44. 10.1186/1743-8977-9-44 PMC3574026 23164071

[64] Nattrass C, Horwell CJ, Damby DE, Brown D, Stone V. The effect of aluminium and sodium impurities on the in vitro toxicity and pro-inflammatory potential of cristobalite. Environ Res. 2017;159:164-75. 10.1016/j.envres.2017.07.054 28802207

[65] Damby DE, Duewell P, Horwell CJ, Baxter PJ, Kueppers U, Schnurr M, Dingwell DB. Volcanic ash activates the NLRP3 inflammasome in macrophages in vitro. Goldschmidt. 2015;648. 10.3389/fimmu.2017.02000 PMC5786523 29403480

[66] Jonasson K (2007). Silicic volcanism in Iceland: Composition and distribution within the active volcanic zones. J Geodyn.

[67] Sommer F, Maschowski C, Dietze V, Grobéty B, Gieré R (2016). Comparing single-particle analysis data of volcanic ash of the 2010 Eyjafjallajökull eruption obtained from scanning electron and light microscope images. Eur J Mineral.

[68] Larsen G, Eiríksson J, Knudsen KL, Heinemeier J (2002). Correlation of late Holocene terrestrial and marine tephra markers, north Iceland: implications for reservoir age changes. Polar Res.

[69] Castro JM, Beck P, Tuffen H, Nichols ARL, Dingwell DB, Martin MC (2008). Timescales of spherulite crystallization in obsidian inferred from water concentration profiles. Am Mineral.

[70] Horwell CJ, Le Blond JS, Michnowicz SAK, Cressey G (2010). Cristobalite in a rhyolitic lava dome: Evolution of an ash hazard. Bull Volcanol.

[71] Baxter PJ, Bonadonna C, Dupree R, Hards VL, Kohn SC, Murphy MD, Nichols A, Nicholson RA, Norton G, Searl A (1999). Cristobalite in volcanic ash of the Soufriere Hills volcano, Montserrat, British West Indies. Science.

[72] Donaldson K, Tran CL (2002). Inflammation caused by particles and fibers. Inhal Toxicol.

[73] Monick MM, Baltrusaitis J, Powers LS, Borcherding JA, Caraballo JC, Mudunkotuwa I, Peate DW, Walters K, Thompson JM, Grassian VH (2013). Effects of Eyjafjallajökull Volcanic Ash on Innate Immune System Responses and Bacterial Growth in Vitro. Environ Health Perspect.

[74] Horwell CJ, Sargent P, Andronico D, Lo Castro MD, Tomatis M, Hillman SE, Michnowicz SAK, Fubini B. The iron-catalysed surface reactivity and health-pertinent physical characteristics of explosive volcanic ash from Mt. Etna, Italy. J Applied Volcanol. 2017;6(1):12.

[75] Fubini B, Mollo L. Role of iron in the reactivity of mineral fibers. Toxicology Letters 1995, 82–83(Proceedings of the International Congress of Toxicology - VII):951–960. 10.1016/0378-4274(95)03531-1 8597167

[76] Pavan C, Rabolli V, Tomatis M, Fubini B, Lison D. Why does the hemolytic activity of silica predict its pro-inflammatory activity? Part Fibre Toxicol. 2014;11:76. 10.1186/s12989-014-0076-y PMC4318150 25522817

[77] Martin E, Sigmarsson O (2007). Crustal thermal state and origin of silicic magma in Iceland: the case of Torfajökull, Ljósufjöll and Snæfellsjökull volcanoes. Contrib Mineral Petrol.

